# Suppression of *Transferrin* Expression Enhances the Susceptibility of *Plutella xylostella* to *Isaria cicadae*

**DOI:** 10.3390/insects11050281

**Published:** 2020-05-05

**Authors:** Huihui Xu, Zhongping Hao, Lifang Wang, Shuangjiao Li, Yuruo Guo, Xiangli Dang

**Affiliations:** 1 Key Laboratory of Biology and Green Control of Plant Diseases and Insect Pests of Anhui Higher Education Institutes, School of Plant Protection, Anhui Agricultural University, Hefei 230036, China; xh15755396709@163.com (H.X.); lsj0926@sohu.com (S.L.); guoyurrrrrrruo@outlook.com (Y.G.); 2 Crop Research Institute, Anhui Academy of Agricultural Sciences, Hefei 230031, China; hzp5187@sina.com; 3 School of Horticulture, Anhui Agricultural University, Hefei 230036, China; wlf2000@ahau.edu.cn

**Keywords:** *Plutella xylostella*, *Isaria cicadae*, transferrin, expression patterns, immunity, development, RNAi

## Abstract

Transferrins (Trfs) are multifunctional proteins with key functions in iron transport. In the present study, a *Trf* (*PxTrf*) from *Plutella xylostella* was identified and characterized. The *PxTrf* consisted of a 2046-bp open reading frame, which encoded a 681 amino acid protein with a molecular weight of 73.43 kDa and had an isoelectric point of 7.18. Only a single iron domain was predicted in the N-lobe of PxTrf. Although *PxTrf* was expressed ubiquitously, the highest levels of expression were observed in the fourth instar larvae. *PxTrf* transcript level was highest in fat bodies among various tissues. The *PxTrf* transcript levels increased significantly after the stimulation of pathogens. A decrease in *PxTrf* expression via RNA interference enhanced the susceptibility of *P. xylostella* to the *Isaria cicadae* fungus and inhibited hemocyte nodulation in response to the fungal challenge. In addition, a considerable increase in the pupation rate was observed in larvae treated with double-stranded PxTrf (dsPxTrf). Overall, according to the results, PxTrf may participate in *P. xylostella* immunity against fungal infection and insect development.

## 1. Introduction

The diamondback moth, *Plutella xylostella* (L.) (Lepidoptera: Plutellidae), is a notorious pest in cruciferous crops and causes an annual economic loss of $4–5 billion [1]. Due to widespread use of insecticides, *P. xylostella* has evolved resistance against various classes of insecticides and some biopesticides, and has become one of the most insecticide-resistant pests globally [2,3]. Therefore, novel *P. xylostella* management strategies are required urgently, and biological control technologies present the greatest potential for the sustainable and efficient control of *P. xylostella* [4,5].

Entomopathogenic fungi are vital group of microorganisms that have been exploited as biological control agents against insect pests [6]. The use of entompathogenic fungi or virulence factors produced by pathogens as pest control is one of the frontiers of biological pest control [7]. However, the effective utilization of the pathogens lies in understanding mechanisms underlying the immune defense responses of host insects to fungi. A pathogenic fungus, *Isaria cicadae*, parasitizes lepidopteran insects, such as *P. xylostella* [8]. To better understand the mechanism of *P. xylostella* immunity, we investigated the immunotranscriptome of *P. xylostella* against *I. cicadae* and identified numerous immunity-related genes. Among immune effector genes, a transferrin (*Trf*) homolog (designated as *PxTrf*) was identified in the *P. xylostella* immunotranscriptome, which exhibited high similarity to insect *Trfs*.

Iron is a key trace element nutrient that participates in various biological processes, such as oxygen and electron transport, ATP generation, cell proliferation, detoxification, gene regulation, and DNA biosynthesis [9,10,11]. In insects, it is also involved in cuticle formation, tanning, melanization, wound healing, and immunity [9]. Due to the adverse effects of excessive or inadequate iron, organisms develop strategies to balance iron concentrations. Numerous proteins, such as transferrins (Trfs), ferritins, iron regulatory proteins, hepcidins, and matriptases, are involved in the transport and metabolism of iron [12]. As a glycoprotein family, Trf family members are the major iron-containing proteins that perform key iron binding and transportation functions [9]. Trfs are widespread in invertebrates and vertebrates. Mammalian Trf and lactoferrin are the two most understood Trf family members [13]. In addition to its iron transport and metabolism functions, mammalian Trf is a growth factor and a transcriptional regulator of gene expression [9]. In addition, lactoferrin plays key roles in the immune system, for example, bactericidal activity and the nuclear factor-κB (NF-κB) activation [14]. Further studies have showed that the Trf family has broad functions and participates in cellular respiration and energy balance [15].

Insect Trf sequences are similar to mammalian Trf and lactoferrin sequences. However, they only retain one iron-binding site in the N-terminal domain [9]. In addition to iron metabolism, insect Trfs have functions in development and stress defense [16,17,18]. Numerous studies have revealed that insect Trfs can also have a role in immunity [9]. *Trf* are upregulated transcriptionally by microbial challenge in numerous insects [19,20,21,22,23]. *Bombyx mori* recombinant Trf protein exhibits an iron binding capacity and antibacterial activity [22]. Iron-free *Manduca sexta* Trf inhibits bacterial growth, whereas iron-saturated Trf exhibits no detectable antibacterial activity [23]. Lehane et al. (2008) reported that the knockdown of *Glossina morsitans Trf* by RNA interference (RNAi) would increase trypanosome infections significantly [24]. Although insect *Trf* has been suggested to have some immune functions, its immunological function during fungal infection has not been explored.

In the present study, we report the characterization and functional analysis of *PxTrf*. *PxTrf* was cloned and its sequence was analyzed using bioinformatics tools. The expression profiles of *PxTrf* in various tissues, at various developmental stages, and under different pathogen challenges, were examined through reverse transcriptase quantitative PCR (RT-qPCR). The immunological functions of *PxTrf* were further explored by RNAi with double-stranded PxTrf (dsPxTrf) injection and subsequent immunological assays in response to *I. cicadae* infection. The efficacy of *PxTrf* on haemocyte nodule formation and larval pupation was also evaluated by injecting dsPxTrf. The results of the present study can enhance our understanding of the roles of *PxTrf* in the immune responses of *P. xylostella* against *I. cicadae* and its potential as a novel target of insect-specific control agents.

## 2. Materials and Methods

### 2.1. Insects and Microorganisms

Diamondback moths, *Plutella xylostella* (L.), were reared on bok choy (*Brassica rapa chinensis*) seedlings at 25 ± 1 °C, 75% ± 5% relative humidity, and a 16:8 h (light/dark) photoperiod. Adults were provided with 10% (w/v) honey solution and allowed to lay eggs on bok choy seedlings.

The Gram-positive bacterium *Staphylococcus aureus* and the Gram-negative bacterium *Escherichia coli* were cultured in Luria–Bertani broth at 37 °C. The entomopathogenic fungus, *Isaria cicadae*, was cultured in potato dextrose agar medium at 28 °C.

### 2.2. cDNA Cloning

Total RNA was extracted from the fourth instar larvae of *P. xylostella* using Trizol reagent (Takara, Beijing, China) according to the manufacturer’s protocol. The quality and concentrations of RNA were evaluated using a NanoDrop 2000 spectrophotometer (Thermo Scientific, Waltham, MA, USA). First-strand complementary DNA (cDNA) was synthesized with 2 μg of total RNA by using the PrimeScript 1st Strand cDNA Synthesis Kit (Takara, Beijing, China). The coding sequence of *PxTrf* was cloned from cDNA by using specific designed primers (Table 1). PCR products were purified using an gel purification of DNA kit (Omega, Bio-tek, Norcross, GA, USA) and sequenced by General Biosystems Corporation Limited, Chuzhou, China).

### 2.3. Sequence Analysis of PxTrf

The *PxTrf* nucleotide sequence was entered into Open Reading Frame Finder (http://www.ncbi.nlm.nih.gov/gorf/gorf.html) to identify open reading frames (ORFs). Signal peptides were predicted using SignalP 5.0 (http://www.cbs.dtu.dk/services/SignalP/), and transmembrane domains of deduced proteins were predicted using TMHMM 2.0 (http://www.cbs.dtu.dk/services/TMHMM/), and conserved domains were predicted using SMART (http://smart.embl-heidelberg.de/). Theoretical isoelectric point (pI) and molecular weight were calculated using PeptideMass (https://web.expasy.org/peptide_mass/). The glycosylation site was predicted using DictyOGlyc (http://www.cbs.dtu.dk/services/DictyOGlyc/). The coiled coil regions were analyzed by COILS (https://embnet.vital-it.ch/software/COILS_form.html). Protein homology modeling was performed using Swiss Model Workspace (https://swissmodel.expasy.org/). PxTrf and its orthologs from other insect species were aligned using DNAMAN 7.0. On the basis of the alignment of whole sequences of insect Trfs, a phylogenetic tree was constructed using MEGA 7.0 using the neighbor-joining method with 1000 bootstrap replications [25].

### 2.4. Reverse Transcriptase Quantitative PCR

RT-qPCR was performed to determine the expression profiles of *PxTrf* at various developmental stages, in different tissues, and under different microbial challenges. Total RNA was extracted separately from the *P. xylostella* eggs, first to fourth instar larvae, pupae, and adults. In addition, total RNA was extracted from the malpighian tubules, epidermis, midguts, fat bodies, and hemocytes of the fourth instar larvae. Furthermore, the fourth instar larvae were treated with *S. aureus* (1.0 × 10^6^ colony-forming unit (cfu)/larva), *E. coli* (1.0 × 10^6^ cfu/larva), and *I. cicadae* spore suspensions (1.0 × 10^7^ cfu/larva). Double-distilled water (ddH_2_O) was used to inject control, and naïve larvae were used as blank controls. Subsequently, they were raised under the conditions mentioned above for 24 h. Each treatment had three independent replicates.

Total RNA isolation and cDNA synthesis were performed using the methods mentioned above. RT-qPCR was performed using SYBR Premix Ex TaqTM II (Takara, Beijing, China) on a Bio-Rad CFX96 (Bio-Rad, Hercules, CA, USA). The qPCR was performed using a mixture (20 μL) of 10 μL SYBR Premix Ex TaqTM II, 0.5 μL of each primer (0.5 μM), 1μL (200 ng) of cDNA, and 8 μL of diethylpyrocarbonate (DEPC)-ddH_2_O. PCR cycling conditions were as follows: denaturation at 94 °C for 5 min, followed by 40 cycles of 95 °C for 10 s, and 60 °C for 20 s. *Actin* was employed as the reference gene. Primers are listed in Table 1. Relative *PxTrf* transcript levels (fold changes) were measured using the 2^−ΔΔCt^ method.

### 2.5. Identification of PxTrf Protein in P. xylostella

Naïve larvae and *I. cicadae* (1.0 × 10^7^ cfu/larvae)-challenged larvae (24 h) were homogenized with precooled phosphate buffer saline (PBS) and centrifuged at 10,000× *g* for 30 min at 4 °C. The supernatants were used for protein identification. PxTrf protein was identified through liquid chromatography–tandem mass spectrometry (LC–MS/MS) according to a previously described method [26]. On the basis of combined MS and MS/MS spectra, protein was successfully identified on the basis of a 95% or higher confidence interval of their scores in the MASCOT V2.3 search engine (Matrix Science Ltd., United Kingdom). The relative quantitation of PxTrf was estimated using the exponentially modified protein abundance index (emPAI) method [27]. The emPAI is defined as follows:

emPAI = 10^PAI^ − 1 (1)

where PAI is equal to the number of observed peptides per protein divided by the number of observable peptides per protein.

### 2.6. RNA Interference

A template corresponding to nucleotides 729–1606 of *PxTrf* for in vitro transcription reactions was prepared through PCR amplification by using gene-specific primers (Table 1). Double-stranded PxTrf (dsPxTrf) was prepared using the TranscriptAid T7 High Yield Transcription Kit (Thermo Scientific, Waltham, MA, USA) according to the manufacturer’s instructions. The reaction mixture included 2 μL 10 × reaction buffer, 8 μL of nucleotide triphosphate (NTP) mixture, 1 μg of the template, and 2 μL of enzyme mix, which was supplemented with nuclease-free water up to a 20 μL volume. After overnight incubation at 37 °C, the reaction mixture was isolated and purified with saturated phenol/chloroform. All synthesized double-stranded RNAs (dsRNAs) were dissolved in nuclease-free water and stored at −80 °C until use. The quantity and purity of dsRNA were determined using a NanoDrop 2000 spectrophotometer (Thermo Scientific, Waltham, MA, USA). The dsGFP corresponding to green fluorescence protein (GFP) was prepared as previously mentioned using the primers (Table 1).

Two microliters of dsPxTrf (5 μg/larva) was injected into the third instar larvae of *P. xylostella* using an IM-31 Microinjector (Narishige Group, Japan). Larvae injected with similar dsGFP amount were considered controls. A DEPC-treated water (2 μL/larva) injection was considered an injection control. After injection, the larvae were raised under the conditions mentioned above. The effects of *PxTrf* silencing at different time intervals (0, 6, 12, 24, 36, and 48 h) were analyzed through RT-qPCR, as described above.

### 2.7. Hemocyte Nodulation Assay

The influence of dsPxTRf treatment on hemocyte nodulation was analyzed using a previously described method [28]. The third instar larvae were injected with dsPxTrf, as described previously. Injection of DEPC-treated water was the injection control, and dsGFP injection was considered a control. Six hours after treatment with dsRNA, the larvae were injected with heat-killed *I. cicadae* spore suspensions (1.0 × 10^8^ cfu/mL). After 10 h of incubation at 25 °C, the nodule numbers were assessed under an Eclipse Ti microscope (Nikon, Tokyo, Japan). Each treatment had three replicates.

### 2.8. Larvae Pupation Analysis

The third instar larvae (30 larvae) were injected with dsPxTrf, as described above. After injection, they were raised under the conditions mentioned above. Pupation rates of dsRNA-treated larvae were assessed at different time intervals (0, 12, 24, 36, and 48 h). Each treatment had three replicates.

### 2.9. I. cicadae Bioassay

The third instar larvae (30 larvae) were injected with dsPxTrf, as described above. After 6 h, larvae were dipped in *I. cicadae* spore suspensions (1.0 × 10^8^ cfu/mL) for 20 s. After infection, larvae were maintained under the conditions mentioned above. Larvae mortality was determined until larvae developed into pupae. Larvae were considered dead when they did not move in response to touch. The experiments were performed three times.

### 2.10. Statistical Analysis

All data are represented as the mean ± standard error. One-way analysis of variance with Duncan’s new repolarization test was performed to compare differences among multiple samples by using the data processing system [29]. The level of significance was set at *p* < 0.05. Survival plot was generated using GraphPad Prism v8.2.1, where *p*-values were determined by Gehan–Breslow–Wilcoxon test.

## 3. Results

### 3.1. cDNA Cloning and Sequence Analysis of PxTrf

**Figure 1:**
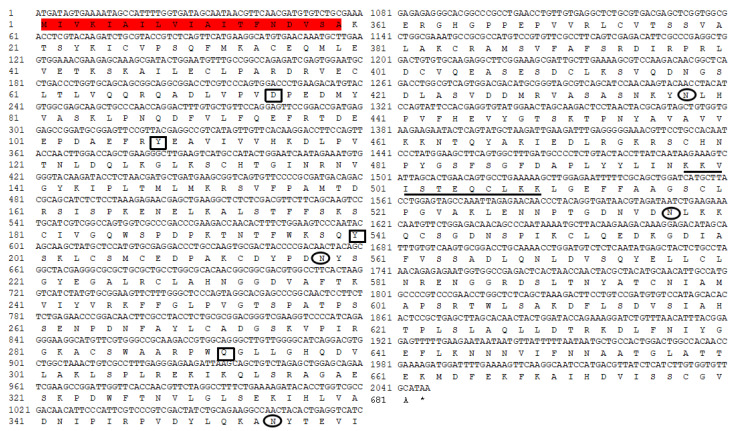
Nucleotide and deduced amino acid sequences of *Plutella xylostella* transferrin (*PxTrf*). Signal peptide is highlighted in red. Iron binding sites are denoted by black rectangles. Putative glycosylation sites are denoted by black oval. The nuclear localization signal is shown by underlining.

A contig (CL847) homologous to insect transferrin was identified in the *P. xylostella* immunotranscriptome against infection by *I. cicadae*. The *PxTrf* cDNA sequence consists of 2254 bp and the open reading frame is 2046 bp (accession no. MN928614), which encodes 681 amino acids (aa) with a 19-aa signal peptide (Figure 1). No transmembrane structure was found in PxTrf. The molecular weight of PxTrf without the signal peptide is estimated to be 73.43 kDa (monoisotopic mass), and the theoretical pI is 7.18. Two conserved Trf motifs (N- and C-lobes) are predicted in each half of the protein. The secondary structure is mostly random coil, 28.93% is the α helix, and 19.24% is the extended strand (Figure S1). PxTrf amino acids are similar to human Trf and lactoferrin (Figure S2). These proteins have two lobes, and the N- and C-lobe of PxTrf are S_22_–V_359_ and V_371_–A_670_, respectively. Only a single iron domain is located in the N-lobe of PxTrf, which includes four conserved active sites (Figure 1). Ten and four conserved cysteines were observed in the N- and C- lobes of PxTrf, and human Trf and lactoferrin, respectively (Figure S2). In addition, four putative glycosylation sites were observed in the Trf sequence, with each lobe having two sites (Figure 1).

**Figure 2:**
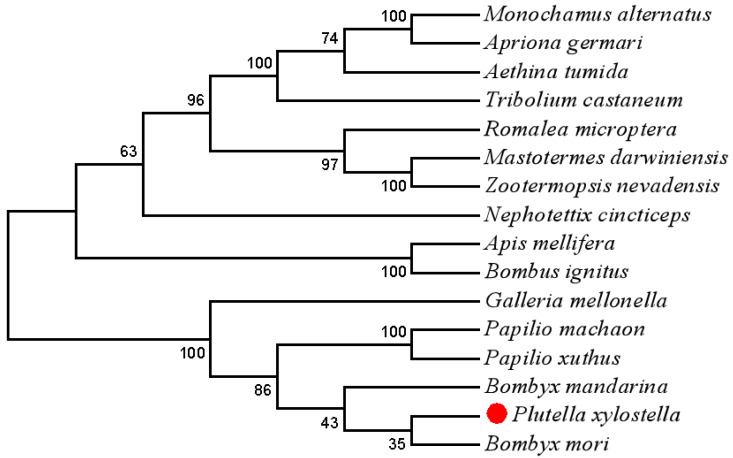
A neighbor-joining phylogenetic tree of *Plutella xylostella* transferrin (Trf) with other insect Trfs using MEGA 7.0. Bootstrap values from 1000 replications are displayed for each branch.

The results of the sequence alignment analysis revealed that most amino acids in the Trf sequence were conserved among the lepidopteran Trfs, particularly in the N-lobe (Figure S3). PxTrf exhibited the highest sequence similarity with *B. mori* Trf. A neighbor-joining phylogenetic tree was constructed to compare the relationship between PxTrf and other insect Trfs (Figure 2). On the basis of the tree, each insect order formed an independent clade, including Coleoptera, Orthoptera, Isoptera, Hemiptera, Hymenoptera, and Lepidoptera. PxTrf was clustered with Trf homologs from lepidopteran insects (such as *Bombyx mandarina*, *B. mori*, *Galleria mellonella*, *Papilio machaon*, and *Papilio xuthus*), and was closely related to Trfs in insects from other *Bombyx* species.

### 3.2. Expression Profiles of PxTrf

**Figure 3:**
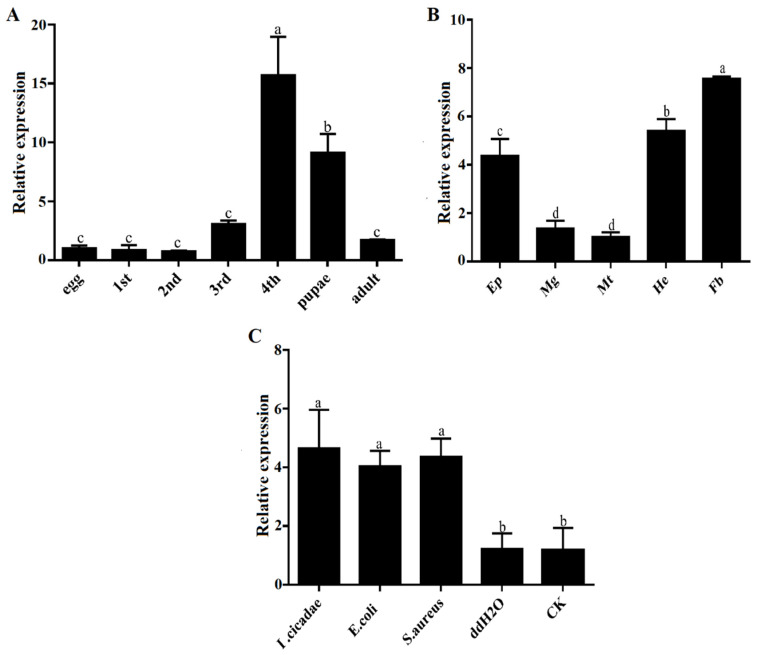
Relative expression levels of *PxTrf*. (**A**) Different developmental stages. Total RNA was extracted from the whole body of *P. xylostella*. (**B**) Different tissues of the fourth instar larvae. Ep: epidermis; Mg: midguts; Mt: malpighian tubules; He: hemocytes; Fb: fat bodies. (**C**) Different microbial challenges of the fourth instar larvae. Data are presented as the mean of three replicates ± standard error. Different lowercase letters indicate significant variations in transcription among different samples (*p* < 0.05).

The *PxTrf* expression patterns at different developmental stages, in different tissues, and under different microbial challenges were determined through RT-qPCR. The highest levels of *PxTrf* transcript were observed in the fourth instar larvae, followed by the pupae, the third instar larvae, and the adults (Figure 3A). *PxTrf* was transcripted at low levels in the eggs, as well as in the first and second instar larvae. All *P. xylostella* tissues expressed *PxTrf*. High transcript levels were found in the fat bodies, epidermis, and hemocytes, with low levels in the midguts and malpighian tubules (Figure 3B). Among various tissues, the highest *PxTrf* transcript levels were observed in the fat bodies. Compared with the control and ddH_2_O groups, *PxTrf* transcript levels were up-regulated significantly after induction by *S. aureus*, *E. coli*, and *I. cicadae* (Figure 3C). The *PxTrf* transcript level after induction by *I. cicadae* was approximately fivefold that of the control.

### 3.3. Identification of PxTrf Protein in P. xylostella

Extracted protein (20 μg) of naïve larvae (Naïve) and *I. cicadae-*challenged larvae (IC) of *P. xylostella* were analyzed through SDS-PAGE (Figure S4) and used for protein identification. According to LC–MS/MS results, PxTrf protein was identified both in Naïve and IC samples. On the basis of the emPAI values, the relative quantitation of PxTrf in the IC sample was 0.52, which was more than twofold that in Naïve larvae (emPAI = 0.23) (Table 2). The result is consistent with *PxTrf* expression levels based on qRT-PCR.

### 3.4. Analysis of the Efficacy of PxTrf RNAi

**Figure 4:**
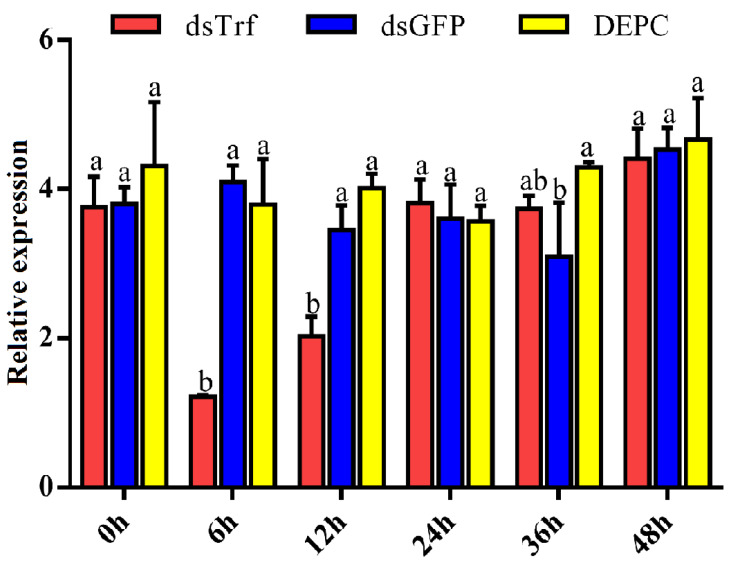
Analyses of the *PxTrf* expression of dsRNA-reated *P. xylostella* using RT-qPCR. dsTrf: injection double-stranded PxTrf (dsPxTrf). dsGFP: injection dsGFP. DEPC: injection DEPC-treated water. Data are presented as the mean ± standard error. Different lowercase letters indicate significant variations in transcription among different samples (*p* < 0.05).

A Trf-specific dsRNA of *P. xylostella* was synthesized in vitro and injected into the third instar larvae. To quantify RNAi efficacy, a RT-qPCR assay was performed. The results showed that compared with the dsGFP (control) and DEPC-treated water (injection control), the *PxTrf* transcription level decreased markedly following dsPxTrf injection (Figure 4). Treatment dsPxTrf markedly suppressed *PxTrf* transcription 6 h post-injection, and the transcript level decreased by 67.8%. The interference effect of *PxTrf* expression decreased gradually with time, reaching a level similar to that in the dsGFP and the DEPC groups at 24 h. dsRNA-treated larvae 6 h post-injection were used in subsequent functional analyses.

### 3.5. Bioassay of PxTrf Silencing P. xylostella to I. cicadae Infection

**Figure 5:**
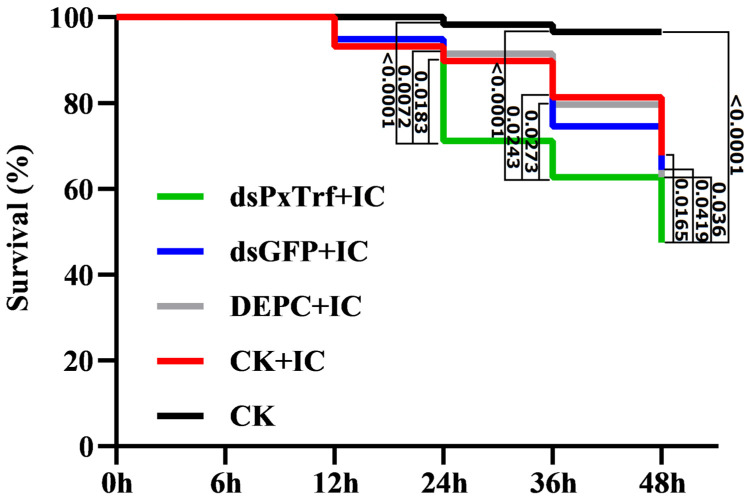
Survival of larvae injected dsRNA following fungal infection. CK: control (uninfection). DEPC: DEPC-treated water injection. dsGFP: dsGFP injection. dsPxTrf: dsPxTrf injection. IC: *I. cicadae* infection. *p*-values are presented only for the treatments with significant difference compared with the dsPxTrf injection treatment. *p*-values were calculated by the Gehan–Breslow–Wilcoxon test.

To determine *PxTrf* function in vivo, *P. xylostella* larvae were infected with *I. cicadae* spore suspensions (1.0 × 10^8^ cfu/mL) 6 h post-dsPxTrf injection. At 48 h post-infection, the survival rate of larvae without any dsRNA injection (CK+IC) was 68.9% (Figure 5). The survival rates of *P. xylostella* DEPC-treated water and dsGFP treated larvae were similar to those of the “CK+IC” group. However, the survival rate of dsPxTrf-treated *P. xylostella* larvae was 48.9% after 48 h of infection, which was significantly lower than that of the control, and those of the DEPC water- and the dsGFP-treated groups. According to the results, injection of dsPxTrf could increase the rate of infection of the fungus *I. cicadae* to *P. xylostella*.

### 3.6. Hemocyte Nodulation Assay

**Figure 6:**
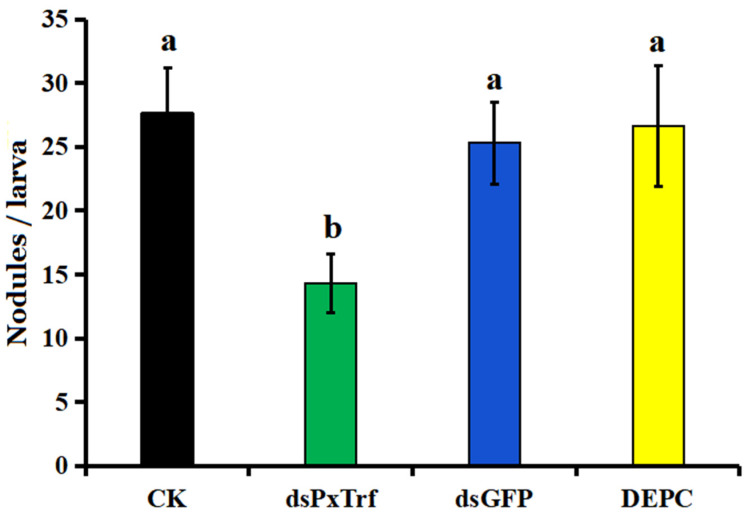
Infuence of dsPxTrf treatment on hemocyte nodulation. CK: without dsRNA injection. dsPxTrf: dsPxTrf injection. dsGFP: dsGFP injection. DEPC: DEPC-treated water injection. Data are presented as the mean ± standard error. Different lowercase letters indicate significant variations among different samples (*p* < 0.05).

In response to *I. cicadae* challenge, the hemocytes of *P. xylostella* without dsRNA treatment (CK) induced around 27.7 nodules per larva (Figure 6). A decrease in *PxTrf* transcription following dsRNA injection impaired nodule formation significantly (*p* < 0.05) in response to fungal challenge. After 10 h incubation at 25 °C, only 14.3 nodules were observed in dsPxTrf-treated *P. xylostella* larvae. No significant differences were observed among CK, DEPC water, and dsGFP groups.

### 3.7. Larvae Pupation Analysis

**Figure 7:**
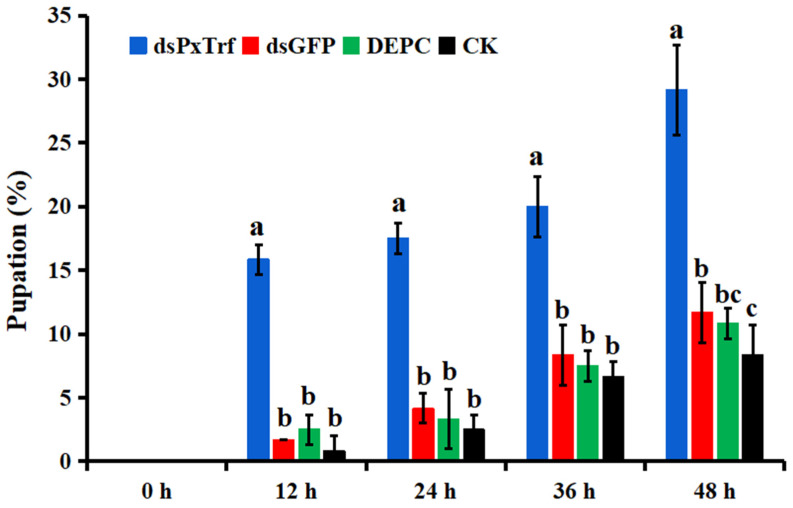
Pupation rate of larvae following dsPxTrf treatment. CK: without dsRNA injection. dsPxTrf: dsPxTrf injection. dsGFP: dsGFP injection. DEPC: DEPC-treated water injection. Data are presented as the mean ± standard error. Different lowercase letters indicate significant variations among different samples (*p* < 0.05).

The efficacy of *PxTrf* RNAi on larval pupation was evaluated in the third instar larvae by the injection of dsPxTrf. The results showed that the pupation rate dramatically increased in dsPxTrf-treated larvae at 12, 24, 36, and 48 h (Figure 7). At 48 h, the pupation rates of the control (CK), DEPC-treated water (DEPC), and dsGFP-treated (dsGFP) groups were 8.3%, 10.8%, and 11.7%, respectively. The pupation rates of dsPxTrf were 29.2% at 48 h, which was significantly higher than the rates in CK, DEPC, and dsGFP treatments.

## 4. Discussion

In the present study, *PxTrf* was identified from the immunotranscriptome of *P. xylostella,* which encodes 681-aa protein and exhibits high sequence similarity with other lepidopteran Trfs. Through bioinformatics analyses, we observed that PxTrf contains a signal peptide without a transmembrane structure. A transferrin gene (*Px-Tf*) (accession no. BAF36818) has previously been predicted from an expressed sequence tag database of *P. xylostella* [28]. Although *PxTrf* and *Px-Tf* encode the same numbers of amino acid residues, they have different molecular weights. In addition, Px-Tf is estimated to be 11.3 pI, whereas the pI of Px-Trf is only 7.18. Usually, insects have more than one *Trf* in their genomes. For instance, there are two *Trf*s in *Drosophila melanogaster*, three in *Tribolium castaneum*, and up to five in *Harpegnathos saltator* [9].

Mammalian Trfs often have 28–38 Cys residues, whereas insect Trfs have 24 or 26 Cys residues [9]. Twenty-four Cys residues were found in the PxTrf sequence (Figure 1). The results of alignment of PxTrf with mammalian lactoferrin and transferrin indicated that highly conserved Cys were mostly in the N-terminal lobe (Figure S2). The conserved Cys in their sequences form intrachain disulfide bonds, which facilitate the maintenance of Trf protein conformations. Disulfide bonds can not only stabilize the protein structure, but are also essential for the binding of iron and Trf receptor action [30].

Structurally, mammalian Trfs usually have two similar domains at the N- and C-terminals, with each containing an iron binding site. The two domains are considered to be formed by gene replication during the evolution process, which can enhance the transport of iron [31]. Although insect Trfs display structures similar to mammalian Trfs (Figure S1), most of them lose the capacity to bind iron at the C-terminal, and only retain the iron binding site at the N-terminal. The N-terminal iron binding residues D, Y, Y, and H/Q are conserved in the N-lobe of PxTrf, mammalian Trf, and lactoferrin (Figure S2). The C-terminal iron binding residues D, Y, Y, and H are completely conserved in the C-lobes of mammalian Trf and lactoferrin, whereas PxTrf lacks both Y residues (Figure S2). *M. sexta* Tsf1 is found to miss both Y and H in the C-lobe and does not exhibit iron binding activity [32]. PxTrf predicted only a single iron domain in the N-lobe. However, Trf of *Blaberus discoidalis* [32], *Mastotermes darwiniensis* [20], and *Protaetia brevitarsis* [33] maintain two iron binding sites in the N- and C-lobes. The function of the C-terminal is the stabilization of the Trf structure and to ensure the iron binding ability [34].

*PxTrf* was found to be highly transcripted in the fat bodies, hemocytes, and epidermis (Figure 3B). In addition, the highest *PxTrf* transcript levels were observed in the fat bodies, which is consistent with the findings of previous studies [18,35,36]. Insect fat bodies and hemocytes are well known to have immune properties, in which various antimicrobial peptides are synthesized in response to microbial challenges [37]. Similar to reports in other insects, *PxTrf* transcript levels increase upon bacterial and fungal challenges, suggesting that *PxTrf* participates in immunity. Notably, PxTrf was also found in naïve *P. xylostella* larvae, and an increase in the relative quantitation of PxTrf was observed in *I. cicadae*-challenged larvae. Brummett et al. (2017) also found that the concentrations of *M. sexta* Trf in the hemolymph increased from 2 to 10 μM following an immune challenge [23]. However, most antimicrobial peptides are not found or are expressed at lower concentration in naïve insects, and are up-regulated considerably following microbial infection [38].

Insect Trf is a multifunctional protein with numerous functions, including iron transport, stress adaptation, immunity, and development [9,39]. As an iron transporter, the iron-binding activity of Trf has been studied extensively in many insects [22,32,40,41,42]. Putative NF-ĸB binding sites are found in the promoter regions of many *Trfs*, suggesting a potential immunological function [22,35,43]. Iron is essential for the host and the pathogen, because both require the element as a cofactor or as a prosthetic group for essential enzymes [44]. The iron-sequestering strategy of host immunity is an effective antimicrobial defense mechanism that combats microbial infection by depriving microorganisms of iron [45,46]. Numerous studies have demonstrated that insect Trfs can restrict the growth of microbial pathogens in vitro [22,23]. Trf also influences bacterial biofilm formation. The addition of 50 μg/mL of human Trf resulted in an 80% decrease in biofilm levels in *Bacillus thuringiensis* [47]. In addition, recombinant human Trf disrupted the membrane potentials of *Acinetobacter baumannii*, *S. aureus*, and *Candida albicans* in a dose-dependent manner [48]. In vivo, insect Trf exhibited an immunological function during infection. *G. morsitans Trf* is upregulated in self-cured flies compared with flies infected with trypanosomes. Furthermore, knockdown of it results in a significant increase in trypanosome infections in the fly midgut [24]. Kim and Kim (2010) found an enhanced susceptibility to infection with a Gram-positive bacterium, *B. thuringiensis*, when *P. xylostella* Tsf1 was silenced [28]. In this study, injection of dsPxTrf could effectively silence *Trf* expression and enhance *P. xylostella* larvae susceptibility to the fungus *I. cicadae*. Furthermore, we observed that a decrease in *PxTrf* expression inhibited hemocyte nodulation significantly in response to *I. cicadae* challenge. Suppression of the *I. cicadae Tsf1* expression also reduced nodule formation in response to bacterial challenge [28]. Therefore, *PxTrf* is potentially involved in *P. xylostella* immunity against fungal infection.

*Trf* is likely involved in insect development [9]. Studies in mammals have shown that *Trf* participates in development [49,50]. Nuclear localization signals are stretches of residues in proteins mediating their importing into the nucleus [51]. A nuclear localization signal was also identified in the PxTrf C-terminal domain (Figure 1), which facilitates Trf regulation of cell growth by interacting with nuclear DNA [28]. We observed *PxTrf* expression at various developmental stages, from eggs to adults. Higher expression levels were observed at the fourth larvae and pupae, and the lower levels occurred at the eggs, and the first and second instar larvae stages (Figure 3A). The pupation rate increased dramatically in larvae treated with dsPxTrf when compared with the control, DEPC water-, and dsGFP-treated larvae. We speculate that this insect adopts the strategy of pupation to adapt to the adverse environmental condition caused by *PxTrf* RNAi. Insect transferrin is regulated by juvenile hormone (JH) in many insects [9]. Generally, JH level is low in insects’ pupae stage [52]. The low level of JH is also found in diapause insects, a physiological state that is used as a means to survive [9]. Zhang et al. (2015) also reported that, compared with the dsGFP control, there was a dramatic body weight decrease in *Helicoverpa armigera* larvae after feeding with Trf dsRNA for 5 days [18]. These findings indicate that *Trf* may participate in insect development.

## 5. Conclusions

In summary, we identified and characterized transferrin from *P. xylostella* and analyzed its multiple functions using RNAi. The highest *PxTrf* transcript levels were observed in the fourth instar larvae and fat bodies. The *PxTrf* transcript levels increased significantly after stimulation with pathogens in vivo. PxTrf protein was identified in both naïve and *I. cicadae*-challenged larvae. Injection with dsPxTrf increased rates of infection of *P. xylostella* with the *I. cicadae* fungus. Furthermore, suppression the *Trf* transcription inhibited nodule formation in response to fungal challenge significantly. In addition, a marked increase in the pupation rate was observed in larvae treated with dsPxTrf compared with the control and the dsGFP-treated larvae. According to the results of the present work, PxTrf can be involved in *P. xylostella* immunity against fungal infection and participate in insect development.

## Figures and Tables

**Table 1:** Nucleotide sequences of primers used in this study.

Primers	Sequence (5′–3′)	Function
*PxTrf-CF*	ATCAGTGACCATGATAGTGAAAATAGCCAT	Cloning
*PxTrf-CR*	GTATGTATGTTTATGCAACACCACAAGATG	
*PxTrf-QF*	TAGCGTCAGCATCCAACAAG	qRT-PCR
*PxTrf-QR*	TCCAAGCTTTTTCAGGCACT	
*Actin-F*	TGGCACCACACCTTCTAC	qRT-PCR
*Actin-R*	CATGATCTGGGTCATCTTCT	
*dsTrf-F*	**TAATACGACTCACTATAGGG**TGTGCGGAAGTTCTTTGGG	RNAi
*dsTrf-R*	**TAATACGACTCACTATAGGG**CTACGTTATCACCTGTAGGGTT	
*dsGFP-F*	**TAATACGACTCACTATAGGG**AGGGCGAGGGCGATGCCACC	RNAi
*dsGFP-R*	**TAATACGACTCACTATAGGG**TGTACTCCAGCTTGTGCCCC	

The bold sequences represent T7 promoter sequence.

**Table 2:** Identification of PxTrf protein in *P. xylostella.*

Sample	Protein	Score	Matches	Sequences	emPAI
Naïve	Transferrin	167	9 (6)	8 (5)	0.23
IC	Transferrin	290	22 (13)	15 (10)	0.52

Score: score of protein identification. Matches: number of matched mass spectra (number with reliability greater than 95%). Sequences: number of matched peptide sequences (number with reliability greater than 95%).

## Supplementary Materials

The following are available online at https://www.mdpi.com/2075-4450/11/5/281/s1, Figure S1: The model structure of PxTrf. The structure was generated by protein homology modeling using Swiss Model Workspace (https://swissmodel.expasy.org/). (A) Human lactoferrin (Protein Data Bank (PDB) ID: 1b0l.1). (B) PxTrf, Figure S2: Alignment of the amino acid sequences of PxTrf with mammalian lactoferrin and transferrin. P: *Plutella xylostella.* H: human (*Homo sapiens*). The N- and C-lobes are indicated by red box and green box, respectively. Active sites are highlighted in yellow. Iron binding sites are denoted by star, Figure S3: Alignment of the amino acid sequences of Lepidopteran Trfs. The N- and C-lobes are indicated by red box and green box, respectively. The conserved amino acids are highlighted in black, Figure S4: SDS-PAGE of extracted protein from *P. xylostella* larvae. IC: *I. cicadae*-challenged larvae. Naïve: larvae without microbial challenge. Mr: protein molecular marker.
